# Comparison of Lipid Profile in Patients With and Without Acute Myocardial Infarction

**DOI:** 10.7759/cureus.6467

**Published:** 2019-12-25

**Authors:** Kheraj Mal, Ratan Kumar, Mishal Ejaz, Kiran Fatima, Faizan Shaukat

**Affiliations:** 1 Cardiology, National Institute of Cardiovascular Diseases, Sukkur, PAK; 2 Cardiology, Khairpur Medical College, Nawabshah, PAK; 3 Internal Medicine, Ziauddin University, Karachi, PAK; 4 Internal Medicine, Jinnah Post Graduate Medical Center, Karachi, PAK

**Keywords:** acute myocardial infarction (ami), lipid profile, pakistan

## Abstract

Introduction

There is ample data available to determine the impact of deranged lipid values of acute myocardial infarction (AMI); however, there is a paucity of data from low-income countries like Pakistan. In this study, we aim to determine the correlation of lipid values with AMI.

Materials and methods

This case-control study was conducted from 1 February 2019 to 30 October 2019 in a tertiary care hospital in Sukkur, Pakistan. There were a total of 421 participants divided into two groups; Case Group (patients with AMI, n=212) and Control Group (patients without AMI, n=209).

Results

Total cholesterol and low-density lipoprotein (LDL) were significantly higher in patients with AMI and HDL was lower. There was no significant difference between triglycerides in both groups.

Conclusion

Dyslipidemia is an important risk factor for AMI. There is a need for more large scale multi-center studies to further understand the role of lipid profile in AMI and the various factors that influence it.

## Introduction

Cardiovascular disease (CVD) is the major cause of mortality and morbidity globally in both males and females [1]. There is evidence available that the Asian population is at greater risk compared to the western world and the majority of this burden falls on the South Asian population [2]. CVD is referred to as a multifactorial disease; it is influenced by both environment and genetic factors. Many well-known factors includes smoking, drinking, diabetes, dyslipidemia and advanced age [3].

In patients with acute myocardial infarction (AMI), dyslipidemia is one of the important risk factors; in particular, abnormal low-density lipoprotein (LDL) and high-density lipoprotein (HDL) are considered an important factor for the development of atherosclerosis and cardiovascular disease [1]. A study conducted in Pakistan, which studied young patients (between 20 to 40 years) with AMI, showed that 60.83% of them were dyslipidemic and that the most common disarranged lipid parameter was triglyceride and the least common was low HDL [4].

Most of the risk assessment for correlation of lipid profile and AMI have been done in developed countries and there is very little data available from low to poor income countries like Pakistan. In this study, we try to address this scientific inequality of data by determining the correlation between AMI and their lipid profile.

## Materials and methods

This case-control study was conducted from 1 February 2019 to 30 October 2019 in a tertiary care hospital in Sukkur, Pakistan. Two hundred and twelve (212) patients with AMI were enrolled during the study period, after informed consent. Two hundred and nine (n=209) controls were also enrolled in the study during this study period. The patients' attendants were used as a control to match the demographic profile. IRB was taken from Nawabshah Medical College.

AMI was confirmed by pertinent history, electrocardiogram (ECG), and cardiac biomarker. Patients with either ST-segment elevation of > 2mm or depression of > 1 mm were included. Troponin and creatinine kinase (CK)-MB were used as biomarkers. Data were collected for variables such as gender, age, smoking history diabetes, hypertension, previous history of myocardial infarction, and family history. Peripheral venous blood samples were collected within 24 hours of admission for measuring lipid profiles. Patients were labelled dyslipidemic according to the Adult Treatment Panel III (ATP III) (Table 1) [5].

**Table 1:** ATP III classification of LDL, total, and HDL cholesterol (mg/dL) ATP III: Adult Treatment Panel III; LDL: low-density lipoprotein; HDL: high-density lipoprotein.

LDL Cholesterol	
< 100	Optimal
100-129	Near Optimal/Above Optimal
130-159	Borderline High
160-189	High
≥ 190	Very High
Total Cholesterol	
< 200	Desirable
200-239	Borderline High
≥ 240	High
HDL Cholesterol	
< 40	Low
≥ 60	High

Statistical analysis was done using Statistical Package for the Social Sciences (SPSS) version 22.0 (IBM Corporation, Armonk, NY). Continuous variables including age and lipid profile were analyzed via descriptive statistics and were presented as mean and standard deviation (SD) while categorical variables including gender, cardiovascular history, smoking history, and the type of lipid abnormality were presented by percentages and frequencies. Means of serum biochemical levels at two-time intervals were compared using paired sample t-test. Categorical data were compared using chi-square. P-value ≤0.05 was taken as significant

## Results

There were a total of 421 participants divided into two groups; Case Group (patients with AMI, n=212) and Control Group (patients without AMI, n=209). The demographics of both groups were compared (Table 1).

**Table 2:** Demographics of participants AMI: acute myocardial infarction.

Demographics	Patients with AMI (n=212)	Patients without AMI (n=209)	P value
Age	56 ± 19	55 ± 19	0.58
Gender Male Female	117 (55.18%) 89 (41.98%)	105 (50.23%) 104 (49.77%)	0.18
Smoking	81 (38.2%)	72 (34.4%)	0.64
Diabetes	37 (17.4%)	30 (14.34%)	0.75
Hypertension	87 (41.03%)	76 (36.36%)	0.32
Previous History of AMI	12 (5.6%)	7 (3.34%)	0.25
Family History of AMI	18 (8.4%)	17 (8,13%)	0.97

Total cholesterol and LDL were significantly higher in patients with AMI. HDL was lower in patients with AMI (Table 2).

**Table 3:** Lipid profiles for the Case and Control groups AMI: acute myocardial infarction; LDL: low-density lipoprotein; HDL: high-density lipoprotein.

Lipid Values (mg/dl(	Patients with AMI (n=212)	Patients without AMI (n=209)	P value
Total Cholesterol	171.48 ± 42.11	160.14 ± 40.17	0.0001
HDL	37.85 ± 12.11	41.23 ± 11.64	0.0004
LDL	94.99 ± 31.97	87.12 ± 31.29	0.02
Triglycerides	145.27 ± 66.31	135.18 ± 60.32	0.1

## Discussion

Dyslipidemia is regarded as a major cause of the excess burden of coronary artery disease among South Asians [6]. Dyslipidemia occurs when there is an increased level of apolipoprotein (apo) B, TG, Lipoprotein Lp(a) and LDL-C, and low levels HDL-C and apoA1 [6]. There are many pieces of evidence suggesting that a decrease in the level of lipoprotein (LDL) levels or an increase in HDL levels can reduce the occurrence of cardiovascular disease [7]. Our study also found that LDL was significantly higher in patients with AMI compared to those without AMI and HDL was significantly lower in patients with AMI compared to those with AMI.

Various trials have indicated that long term exposure to lower levels of LDL can reduce the risk of cardiac events [8-9]. In this study, LDL was higher in participants with AMI compared to those without AMI (94.99 ± 31.97 mg/dl vs 87.12 ± 31.29). In a large scale meta-analysis, covering almost 170,000 individuals, it was concluded that treatment with statins was associated with a log linear proportional reduction of 22% in major cardiovascular events for every per millimole per litre reduction in LDL-C over a median of five years of treatment [10].

In this study, HDL was significantly lower in patients with AMI than compared to those without AMI. There are contrary data available on the impact of HDL on cardiovascular disease. Some studies have established an inverse relationship between HDL level and cardiovascular disease [11], while Mendelian randomization argues against the causal relation between HDL and cardiovascular disease [12].

In this study, there was no significant difference between triglyceride (TG) levels of patients with AMI and patients without AMI. Emerging risk collaboration in their study concluded that increased level of plasma TG levels are associated with an increased risk of ASCVD, however, after adjusting for non-HDL-C this association becomes null [11].

It is also important to understand the role of lipoprotein in atherosclerosis and cardiovascular disease. All ApoB-containing lipoprotein with a diameter smaller than 70 mm can cross the endothelial barrier, particularly when there is endothelial dysfunction. They get trapped after interaction with extracellular structures. These ApoB-containing lipoproteins provoke a complex process that leads to lipid deposition and the initiation of an atheroma. Continuous exposure to Apo B-containing lipoprotein may lead to an increase in particles that are retained over time in the walls of the artery and causes continuous growth and progression of atherosclerotic plaque. People with a higher concentration of plasma ApoB-containing lipoproteins will retain more particles and accumulate lipids faster, resulting in more rapid growth and the progression of atherosclerotic plaques.

Despite being an important factor, there are very limited studies about the role of dyslipidemia in patients with myocardial infarction. Our study, to the best of our knowledge, is first from our region of rural Sindh. However, there are a few limitations as well. This study was single-center study, hence its result cannot be generalized to an entire population While we tried our level best to match both the Case and Control Group, there were few confounding factors which were not noted due to the critical condition of patients such as body mass index (BMI). Patients outcomes were also not noted.

## Conclusions

In this study, total cholesterol and LDL were significantly higher in patients with AMI. HDL was lower in patients with AMI. National consensus on periodic screening of lipid profile should be made with the efforts of health care professionals and the government. More large scales studies should be conducted to understand the role of various lipoproteins and their correlation with CVD.
